# Hypertrophic Obstructive Cardiomyopathy Masked by Tako-Tsubo Syndrome: A Case Report

**DOI:** 10.1155/2012/486427

**Published:** 2012-03-21

**Authors:** Y. Daralammori, M. El Garhy, M. R. Gayed, A. Farah, B. Lauer, M. A. Secknus

**Affiliations:** ^1^Department of Cardiology, Heart Center, Zentralklinik Bad Berka, Robert-Koch-Allee 9, 99437 Bad Berka, Germany; ^2^Department of Cardiology, Minia University, Kornish El Nil Street 6111, Minia, Egypt

## Abstract

*Introduction*. Left ventricular outflow obstruction might be part of the pathophysiological mechanism of Tako-tsubo cardiomyopathy. This obstruction can be masked by Tako-tsubo cardiomyopathy and diagnosed only by followup. *Case Presentation*. A 70-year-old female presented with Tako-tsubo cardiomyopathy and masked obstructive hypertrophic cardiomyopathy at presentation. *Conclusion*. Tako-tsubo cardiomyopathy typically presents like an acute MI and is characterized by severe, but transient, regional left ventricular systolic dysfunction. Prompt evaluation of the coronary status is, therefore, mandatory. The prognosis under medical treatment of heart failure symptoms and watchful waiting is favourable. Previous studies showed that LVOT obstruction might be part of the pathophysiological mechanism of TCM. This paper supports this theory. However, TCM may also mask any preexisting LVOT obstruction.

## 1. Introduction

Tako-tsubo cardiomyopathy (TCM) is an acute cardiac syndrome of unknown etiology characterized by severe but transient systolic dysfunction of the apical and/or mid segments of the LV mimicking myocardial infarction in the absence of obstructive coronary artery disease [1, 2]. This form of contractile dysfunction is typically transient and reversible within days or weeks [3, 4]. Symptoms are similar to those of acute MI, including sudden onset of chest pain associated with convex ST-segment elevation and a moderate increase in creatine kinase and troponin levels [5]. Symptoms commonly occur after emotional or physical stress [3, 5, 6], predominantly in postmenopausal women (90% of cases) [3, 7, 8]. An association with malignancies has been reported in approximately 50 patients, potentially as a result of paraneoplastic phenomena [9, 10]. 

Several studies showed that left ventricular outflow tract obstruction (LVOTO) might be present in up to 25% of patients with TCM. It remains unclear if LVOTO is the cause or result of TCM. There are a few case reports in the literature reporting an association between TCM and hypertrophic obstructive cardiomyopathy (HOCM). In these patients, there was a pressure gradient below the level of the aortic valve between the aorta and the left ventricle. 

## 2. Case Presentation

**Figure 1:**
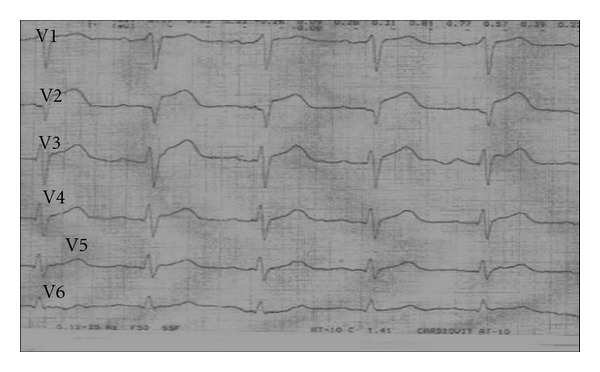
Chest leads electrocardiogram showing ST-segment elevations in V2, V3, and V4.

**Figure 2:**
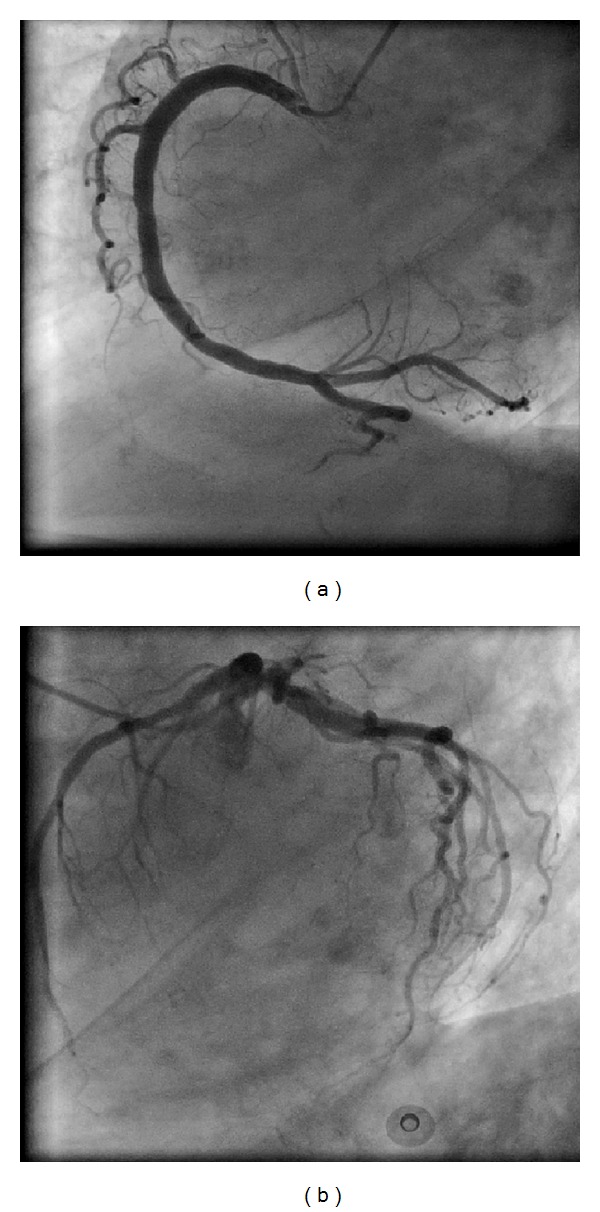
Coronary angiogram minor disease in the left anterior descending artery but no other coronary artery disease.

**Figure 3:**
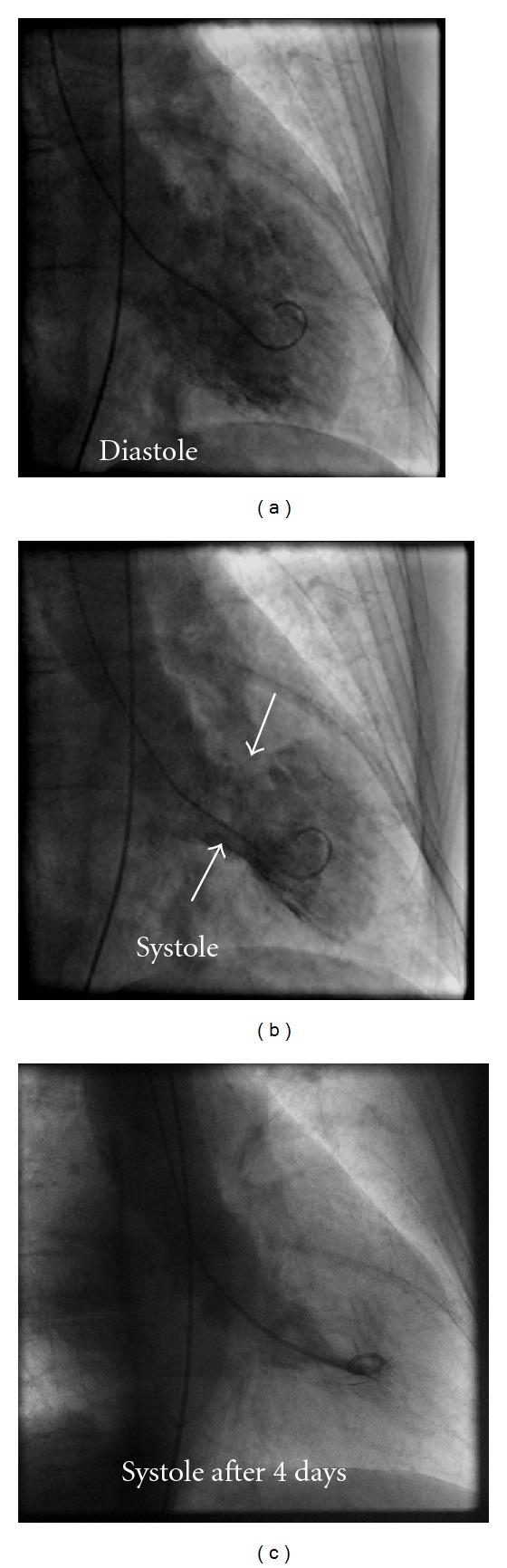
LV angiogram in diastole (a) and systole (b) in right anterior oblique projection demonstrating wall-motion abnormality characteristic of stress cardiomyopathy. At end systole, LV chamber adopts distinctive “short neck with round flask” configuration in which distal (apical) portion is akinetic/hypokinetic, whereas in contrast, the remaining proximal (basal) segment is hypercontractile. Left panel, angiogram after 4 days.

**Figure 4:**
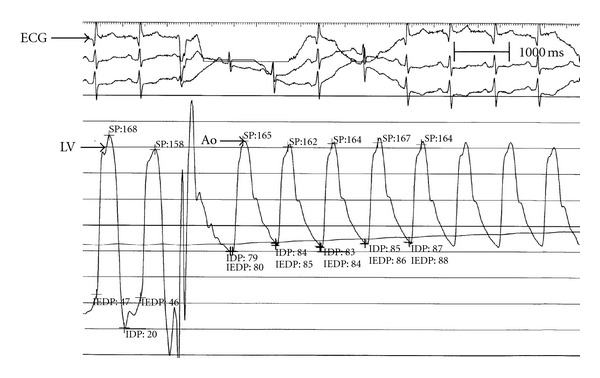
Pressure tracings show a sharp rise in LV outflow gradient that follows the pause associated with PVC. A dynamic obstruction leads to a concomitant fall in aortic pressure and a disproportionate (46 to 130 mmHg) increase in gradient. This phenomenon, known as the Brockenbrough-Braunwald-Morrow sign, is part of the classical description of hypertrophic obstructive cardiomyopathy. Ao: aorta; ECG: electrocardiogram; LV: left ventricle; PVC: premature ventricular complex.

A 70-year-old female patient presented to the emergency room complaining of sudden onset shortness of breath. Past medical history was noncontributory except for hypertension. Patient's vital signs included: blood pressure 160/80 mmHg, resting heart rate 84 beats/min, respiratory rate 18 breaths/min, oxygen saturation 95%, and temperature 37.0°C. Cardiac auscultation revealed normal first and second heart sounds and no murmurs. Jugular venous pressure was normal. Neither lower limb edema nor signs of pulmonary congestion were noticed. The initial ECG showed ST-elevation in the precordial leads from V2 to V4 (Figure 1). The initial diagnosis of acute coronary syndrome (ST elevation MI) was established, and the patient was immediately transferred to our cardiac catheter lab. Coronary angiogram, however, showed some atherosclerotic coronary artery disease, but no significant stenosis (Figure 2). Left ventriculography demonstrated typical apical ballooning with a globally reduced ejection fraction estimated at 35% (Figure 3). Pressure tracings showed no pressure gradient between the LV and the aorta (Figure 4).

The patient's complete blood count, basic metabolic panel, and liver function tests were all within normal range. Two sets of myocardial enzyme assays showed a progressive increase in creatine phosphokinase from 2.1 *μ*mol/s/L to 3.1 *μ*mol/s/L (normal range < 2.4 *μ*mol/s/L), and troponin I from 2.04 ng/mL to 5.89 ng/mL (normal range 0–0.15 ng/mL) during the first 6 hours after admission. The patient was transferred to ICU and stabilized by standard medical heart failure management including beta blockers, diuretics, and ACE-inhibitors.

After 3 days, echocardiography revealed normal LV systolic function, no resting wall motion abnormalities, LV septal wall thickness of 24 mm, and systolic anterior motion (SAM) of the anterior mitral valve leaflet. There was a peak systolic pressure gradient below the level of aortic valve of 20 mmHg at rest, increasing to 70 mmHg after Valsalva maneuver.

**Figure 5:**
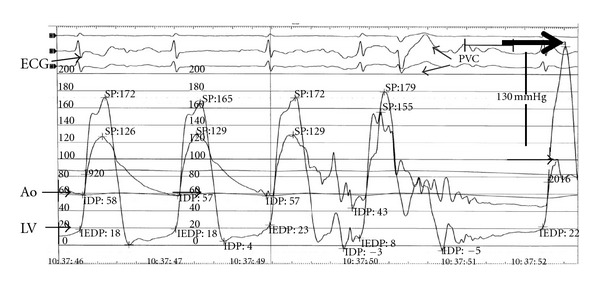
Pressure tracings show a sharp rise in LV outflow gradient that follows the pause associated with PVC. A dynamic obstruction leads to a concomitant fall in aortic pressure and a disproportionate (46 to 130 mmHg) increase in gradient. This phenomenon, known as the Brockenbrough-Braunwald-Morrow sign, is part of the classical description of hypertrophic obstructive cardiomyopathy. Ao: aorta; ECG: electrocardiogram; LV: left ventricle; PVC: premature ventricular complex.

On day four, a second invasive hemodynamic evaluation was performed, confirming a simultaneous pressure gradient between the LV and aorta of 45 mmHg at rest that increased to 70 mmHg after Valsalva maneuver (Figure 5). Pressure tracings of a postpremature ventricular contraction (PVC) beat showed a sharp rise of the LVOT gradient to 130 mmHg (Brockenbrough-Braunwald-Morrow sign), which is part of the classical description of hypertrophic obstructive cardiomyopathy [11]. 

The patient was discharged on medical treatment including beta blocker, statins, and low dose aspirin. Upon followup after 3 months, the patient was asymptomatic whereas transthoracic echocardiography continued to show the same gradient of 50 mmHg across the LVOT.

## 3. Discussion

Tako-tsubo cardiomyopathy, also known as stress cardiomyopathy, transient apical ballooning, or broken heart syndrome, has raised interest since it was first described by Dote et al., who named it Tako-tsubo since the shape of the LV resembles a Japanese octopus trap, with a round bottom and narrow neck [1].

The prevalence of TCM among patients with symptoms suggestive of acute MI is 0.7–2.5% [3, 7, 8]. In-hospital mortality rate is 2% [9]. The prognosis is favourable [3] although fatal complications, such as cardiogenic shock, pulmonary edema, LV thrombus formation, malignant arrhythmias, free wall rupture of the LV, and death have been reported [12–15]. Patients with TCM after recovery have a 4-year cardiovascular survival similar to people from the general population matched for age and sex [9, 10].

There are no controlled data to define the optimal medical regimen of the disease. It appears reasonable to treat these patients with standard medications for LV systolic dysfunction including ACE-inhibitors, beta blockers, diuretics, inotropes, and intra-aortic balloon counterpulsation if needed [6, 16, 17].

The exact etiology of TCM is still unknown, but several theories have been proposed. These include multivessel coronary artery spasm, obstruction of the LVOT, impaired cardiac microvascular function, impaired myocardial fatty acid metabolism, acute coronary syndrome with reperfusion injury, and endogenous catecholamine-induced myocardial stunning and microinfarction [2, 18–21].

Exposure to endogenous (e.g., emotional) or exogenous (trauma, surgical procedure) stress and increased sympathetic activity have been reported in most cases of TCM. This association suggests that the mechanism of disease might be sympathetically mediated [18]. Plasma catecholamine concentrations were around two to three times higher in patients with TCM compared to patients hospitalized for acute MI (Killip class III) [20]. Excessive catecholamine release in pheochromocytoma is known to induce reversible LV dysfunction [22]. TCM is characterized by microscopic morphological alterations similar to those following catecholamine cardiotoxic effects reported in animals and humans [23, 24].

Early studies showed that LVOT obstruction might be present in 25% of patients with TCM. Echocardiography reveals a typical septal bulge associated with systolic anterior motion of the mitral valve and mitral regurgitation similar to the findings associated with HOCM [25]. Additional reports have confirmed structural abnormalities associated with LVOT obstruction, such as mid-ventricular septal thickening. This feature could potentially cause severe, transient LV mid-cavity obstruction in the presence of increased catecholamine levels [19]. It remains uncertain if LVOT obstruction is a result or cause of stress cardiomyopathy [18].

There are very few case reports about TCM in patients with hypertrophic obstructive cardiomyopathy [11]. They all report a pressure gradient between the aorta and apical LV upon clinical presentation. In our case, the reverse had occurred; there was no pressure gradient between the aorta and apical LV during the acute attack. The gradient only (re-)appeared after improvement of LV systolic function. Two possible explanations for this gradient masking have to be considered. During the acute attack, there might be severe systolic myocardial impairment with subsequent low flow phenomenon masking the gradient. This picture is similar to the scenario of patients with severe aortic stenosis but low aortic valve pressure gradients due to LV systolic impairment. Another explanation might include the involvement of the basal septum in the pathology of TCM resulting in a transient pseudonormalization of the systolic pressure gradient.

## 4. Conclusions

Tako-tsubo cardiomyopathy typically presents like an acute MI and is characterized by severe, but transient, regional left ventricular systolic dysfunction. Prompt evaluation of the coronary status is, therefore, mandatory. The prognosis under medical treatment of heart failure symptoms and watchful waiting is favourable.

Previous studies showed that LVOT obstruction might be part of the pathophysiological mechanism of TCM. This paper supports this theory. However, TCM may also mask any preexisting LVOT obstruction.
